# Neutrophil Extracellular Traps and By-Products Play a Key Role in COVID-19: Pathogenesis, Risk Factors, and Therapy

**DOI:** 10.3390/jcm9092942

**Published:** 2020-09-11

**Authors:** Alain R. Thierry, Benoit Roch

**Affiliations:** 1 Research Institute of Cancerology of Montpellier, INSERM U1194, IRCM, ICM, Montpellier University, F-34298 Montpellier, France; 2 Respiratory Medicine, University Hospital of Montpellier, Montpellier University, F-34298 Montpellier, France; benoitroch.med@gmail.com

**Keywords:** COVID-19, neutrophil extracellular traps, circulating DNA, therapy, risk factors, patho-physiology

## Abstract

Understanding of the pathogenesis of the coronavirus disease-2019 (COVID-19) remains incomplete, particularly in respect to the multi-organ dysfunction it may cause. We were the first to report the analogous biological and physiological features of COVID-19 pathogenesis and the harmful amplification loop between inflammation and tissue damage induced by the dysregulation of neutrophil extracellular traps (NETs) formation. Given the rapid evolution of this disease, the nature of its symptoms, and its potential lethality, we hypothesize that COVID-19 progresses under just such an amplifier loop, leading to a massive, uncontrolled inflammation process. Here, we describe in-depth the correlations of COVID-19 symptoms and biological features with those where uncontrolled NET formation is implicated in various sterile or infectious diseases. General clinical conditions, as well as numerous pathological and biological features, are analogous with NETs deleterious effects. Among NETs by-products implicated in COVID-19 pathogenesis, one of the most significant appears to be elastase, in accelerating virus entry and inducing hypertension, thrombosis and vasculitis. We postulate that severe acute respiratory syndrome-coronavirus 2 (SARS-CoV2) may evade innate immune response, causing uncontrolled NETs formation and multi-organ failure. In addition, we point to indicators that NETS-associated diseases are COVID-19 risk factors. Acknowledging that neutrophils are the principal origin of extracellular and circulating DNA release, we nonetheless, explain why targeting NETs rather than neutrophils themselves may in practice be a better strategy. This paper also offers an in-depth review of NET formation, function and pathogenic dysregulation, as well as of current and prospective future therapies to control NETopathies. As such, it enables us also to suggest new therapeutic strategies to fight COVID-19. In combination with or independent of the latest tested approaches, we propose the evaluation, in the short term, of treatments with DNase-1, with the anti-diabetic Metformin, or with drugs targeting elastase (i.e., Silvelestat). With a longer perspective, we also advocate a significant increase in research on the development of toll-like receptors (TLR) and C-type lectin-like receptors (CLEC) inhibitors, NET-inhibitory peptides, and on anti-IL-26 therapies.

## 1. Introduction

December 2019 saw the emergence of severe acute respiratory syndrome-coronavirus 2 (SARS-CoV2) which causes coronavirus disease-2019 (COVID-19), an acute respiratory disease [1]. The Center for Disease Control and Prevention (CDC) reports illnesses ranging from mild symptoms to severe illness and death for confirmed cases. Incubation lasts on average five days, with extremes of two to twelve days, the onset of symptoms appearing gradually with headache, myalgia, fatigue, fever, cough, dyspnea, and chest pain. The World Health Organization (WHO) distinguishes several clinical syndromes associated with SARS-CoV2: asymptomatic forms, uncomplicated disease, non-severe pneumonia and severe pneumonia, acute respiratory distress syndrome (ARDS), i.e., life-threatening respiratory insufficiency, sepsis and septic shock with multi-organ failure syndrome.

Infection-related conditions such as viral, bacterial or mycotic pneumonia may directly injure the lungs and cause acute lung injury (ALI) or ARDS. ALI is described as a lung disease with acute onset and disruption of the alveolar-capillary interface, leading to increased microvascular permeability. ALI and ARDS can have many different causes, but epithelial injury is the basis of ARDS, which is a more severe form of ALI. We propose to correlate ARDS/ALI with innate immunity in respect to COVID-19.

Here we review the possible links between COVID-19 pathogenesis and NET dysregulation. We include an overview of NET formation, function and pathogenic dysregulation, as well as existing and future therapies designed to control NET unbalance and its potential adverse effects (NETopathies). Because of the complexity of both COVID-19 and of NET formation, which involve a great number of pathways and signaling, we have arbitrarily described in detail the common biological and physiological features of both as currently understood. Our objective is subsequently to propose new strategies for treating the pandemic.

## 2. Innate Immunity

Cells participating in innate immunity represent the body’s first line of defense against invading microbes, being responsible for neutralizing those invaders, and for steering the organism’s adaptive immunity in case of persistent infection [2]. Given the growing evidence of the part they play in antiviral immune responses, neutrophils might be considered the main ‘foot soldiers’ constituting such innate immunity.

The generally accepted understanding is that neutrophils fight against micro-organisms by directly phagocytosing their targets, or by releasing toxic components via degranulation. Even so, it should be noted that neutrophils also indirectly defend the host against microbes by participating in the elaboration of cell signaling networks involving cytokines, chemokines, survival and growth factors that cause downstream pro-inflammatory effects. Typically, neutrophils are found in higher concentrations in the pulmonary capillaries, as compared to systemic blood, even in the absence of inflammatory stimuli. This phenomenon allows neutrophils to readily migrate into the lungs in response to inflammatory stimuli. Neutrophils undergo cellular deformation in order to perform a transendothelial migration from pulmonary capillaries to alveolar air spaces. Neutrophil activating factors may be derived from the host (e.g., platelet activating factor (PAF), leukotriene B4, interleukin-8 (IL-8 or CXCL8)), or from pathogens (e.g., formylated peptide and lipopolysaccharide (LPS)).

Over the last 25 years, the importance of cell death pathways and the way in which immune cells respond to dead and dying cells has been intensively studied, and their importance in many diseases demonstrated. In this context, an additional antimicrobial function of neutrophils should be noted, relying on a special type of programmed cell death called NET formation [3]. In response to injury, neutrophils are able to generate threads of chromatin covered with granule-derived peptides and (proteolytic) enzymes, namely neutrophil extracellular traps (NETs) [4]. These virus-induced NETs can control the virus by means of their large net-like structure, in which the pathogens get trapped [5].

## 3. NET Formation and Extracellular DNA/Nucleic Acids Release

NETs were discovered in 2004 [6], and have been described as a potential bacterial killing mechanism. Brinkmann et al. [6] were the first to report the way in which neutrophils are activated to release their deoxyribonucleic acid (DNA), which is laden with proteases that traps and kills microbes [7].

### 3.1. NET Formation/General Function

One of the neutrophil’s effector mechanisms is NET formation, which allows the release of NETs in the extracellular medium. These NETs correspond to extracellular filaments of uncondensed chromatin (an association of DNA and histones) covered by numerous proteins of mainly granular origin.

NETs have been linked with severe infections, such as sepsis, and serves as an additional defense of the innate immune system against circulating microbes, including bacteria, fungi, protozoa, and viruses. Conservation of the NET function across species suggests an evolutionary advantage of NETs in immune defense. The formation of NETs was originally described as a new cell death program, different from apoptosis and necrosis.

This “classic” NET formation is dependent on the oxidative explosion, and leads to the release of NETs by 20 to 60% of human neutrophils, after 2 to 4 h of stimulation with microorganisms or activators of protein kinase C (PKC), such as phorbol myristate acetate (PMA). During this process, the histones are cleaved by the elastase derived from the granulations, and citrullinated by peptidyl arginine deiminase 4 (PAD4). This process leads to decondensation and sagging of the chromatin. The combined rupture of the nuclear and granular membranes leads to the mixing of the cytoplasmic, granular and nuclear components. The rupture of the plasma membrane then allows the release of NETs in the extracellular space. Initially, neutrophil elastase degrades the linker histone protein H1 and the core histone protein, resulting in chromatin decondensation, which is enhanced by myeloperoxidase (MPO). A recent proteome analysis showed that the main components of NETs are DNA, elastase and histones H1, H2A, H2B, H3, and H4 [8]; and other components including: neutrophil elastase (NE), MPO, bactericidal/permeability-increasing protein, cathepsin G, lactoferrin, matrix metalloproteinase-9, peptidoglycan recognition proteins, pentraxin, and LL-37 [9]. While extracellular traps (ETs) formation is prominent in neutrophils, several other types of innate or adaptative immune cells reportedly release, following strong activation signals, chromatin and granular proteins (MPO, NE, etc.) into the extracellular space, forming ETs: macrophages, eosinophils, basophils, mast cells and lymphocytes [10,11].

### 3.2. Stimuli

The stimuli which induce polymorphonuclear neutrophils (PN) NET formation can vary. They include many micro-organisms and their components (LPS, lipopolysaccharide), pro-inflammatory cytokines (IL-8, tumor necrosis factor α (TNF α)), activated platelets, or immune complexes [12]. While the molecular mechanisms leading to NET formation have yet to be fully established, various studies have given rise to a “classic” model involving PN cell death. “Alternative” NET formation mechanisms, depending in particular on the trigger stimulus that do not involve cell death, exist.

In the ‘classic’ model, modification by citrullination of histone H3 is thought to be involved in the in vitro formation of NETs. Hirose et al. [9] have described the relationships between the presence of NETs and citrullinated histone H3 (Cit-H3) caused by the existence of bacteria in tracheal aspirate, systemic inflammatory response syndrome (SIRS) diagnosis, white blood cells (WBC) count, and concentrations of IL-8, TNF-α, circulating DNA (cirDNA), lactate, and high mobility group box-1 (HMGB1). NET-inducing factors identified to date include IL-8, PMA, bacteria, mycobacteria, fungi, protozoa, PAF, LPS, and M1 protein. Classic NET formation requires the generation of reactive oxygen species (ROS) by NADPH oxidase. However, mitochondrial ROS production in the absence of a functional NADPH oxidase is sufficient to trigger NET formation. ROS act as key signalling molecules for NET formation independent of the type of stimulus and granulocyte. In order to maintain homeostasis, NETs are then degraded by the combined action of DNase-1 and an endocytosis mechanism involving macrophages.

In contrast to this “classic” model, several studies have reported a phenomenon of rapid NET formation (5–60 min) in different experimental models. The cellular mechanism is variable, but none of these rapid NET formation causes the rupture of the plasma membrane, nor the death of the PN. Some involve the release of mitochondrial DNA (by stimulation, through the association of complement protein 5a (C5a) and granulocyte-macrophage colony-stimulating factor (GM-CSF). Others use an extracellular release of vesicles containing nuclear DNA notably stimulated by Staphylococcus aureus. A recent study even showed that, after NET formation, PN retain their ability to migrate and to perform phagocytosis. This ability is associated with the release of neutrophil-derived enzymes. This alternative rapid mechanism would explain the role of NET formation in various acute phenomena [13].

### 3.3. Role of Platelets

The interplay of neutrophils and platelets potentializes an innate immune response. NET formation involves platelets being sequestered in the lung microcirculation in a neutrophil-dependent process. In preclinical studies, Caudriller et al. [14] have shown that platelet depletion protects mice from severe lung injury and mortality. Platelets promote inflammation and injury through critical interactions with neutrophils. For instance, in sepsis, the activation of platelet TLR4 is a potent stimulus for the release of NETs by neutrophils residing in the sinusoids of the liver and in other capillary beds. The same study observed ultrastructural evidence of neutrophil-platelet aggregates in the lung microcirculation which included close interactions between the lung endothelium and red blood cells. The innate immune postulated function of NETs resides in skimming plasma for intravascular pathogens; other intravascular components, nonetheless, may also be efficiently trapped, causing detrimental effects. Notably, an intense platelet sequestration in areas of NET formation has been observed [14]. The high binding of platelets to NETs is mediated through interactions with charged extracellular histones. Thus, platelet accumulation on NETs extends platelet aggregation and activation, promoting coagulation in the lung microcirculation, which may lead to ischemic consequences.

## 4. NET Nucleotidic Components: Nucleosomes and Circulating Cell-Free DNA

### 4.1. Nuclear DNA

Nucleic acids appear to be the main source of damage-associated molecular patterns (DAMPs), and stimulate pattern recognition receptors (PRR) to induce inflammation [3]. The location of nucleic acid sensing receptors can be conveniently classed as either endosomal or cytosolic. They are recognized by DNA sensors such as TLR9, and by stimulator of interferon genes (STING), which are secluded in endosomes and cytosol, respectively; this recognition triggers type I interferon and proinflammatory cytokine secretion, thereby controlling host defense countermeasures. TLR9 is mainly expressed by B lymphocytes and plasmacytoid dendritic cells (pDCs) in humans, and recognizes DNA containing unmethylated CpG oligodeoxynucleotide motifs which are more prevalent in microbes. STING, expressed in numerous cell types (including myeloid and lymphoid cells) recognizes a wide variety of DNA [3]. Microbial DNA constitute potent microbial moieties that activate innate immune cells. While high molecular weight DNA is found circulating in blood, most is highly fragmented [15,16], and mainly in the form of mononucleosomes [17,18,19]. Circulating DNA in the form of nucleosomes has been reported to correlate with organ dysfunction, disease severity and mortality in sepsis patients and children suffering from meningococcal sepsis.

Under pathologic conditions, extracellular DNA and histones may be part of an amplifier-loop between inflammation and tissue damage. Necrosis attracts neutrophils, which in turn exacerbate tissue damage. In vitro experiments suggest that NETs, mononucleosomes or extracellular histones contribute to the cytotoxicity [20]. The role of nucleosome in autoimmune diseases was demonstrated, as well as histones antimicrobial activity, yet can also cause tissue damage and other pathological abnormalities. Thus, histones released from NETs are, by themselves, unique cytotoxic DAMPs linking cell necrosis and inflammation (as reviewed by Nakasawa et al., 2018 [21]).

### 4.2. Mitochondrial DNA

Infections by bacteria, fungi, and parasites cause the release of nuclear DNA as well as abundant mitochondrial DNA (mtDNA). Various studies have provided convincing evidence of the role of mitochondria in NET formation, and of their possible role as a DAMP. Yousefi et al. [2] identified mitochondria as a source of scaffold DNA for extracellular traps. Interestingly, all types of granulocytes, neutrophils, eosinophils, and basophils release mtDNA to form extracellular traps, which then bind and kill bacteria in the absence of detectable cell death. In addition, oxidized mtDNA is highly inflammatory, as it activates the STING pathway, inducing IFN-β and other inflammatory cytokines [3]. Mitochondria are readily damaged through hyper- or hypo-polarization of their membranes and by the generation of ROS. Elkon et al. [3] demonstrated that once oxidized, mtDNA is less efficiently degraded by DNases such as TREX1, leading to activation of DNA sensors [22].

As recently reviewed by [23], viruses appear to influence microbial pathogenesis by manipulating mitochondria or their constituents. Several viruses (dengue virus, human immunodeficiency virus (HIV) and SARS) induce the mitochondrial fusion required for intracellular proliferation of the virus and evasion of the antiviral innate immune signaling. Moreover, open reading frame 9b (ORF-9b), a virulence factor of SARS-CoV, induces proteasomal degradation of dynamin-related protein 1 (DRP1), thereby leading to mitochondrial fusion and fragmentation, which eventually limits host cell inducible interferon (iIFN) responses against the virus. Furthermore, mtDNA and the mitochondrial lipid cardioliopin act as potent activators of the NLRP3 (NOD-, LRR- and pyrin domain-containing protein 3) inflammasome. Nucleotide-binding oligomerization domain (NOD)-like receptors (NLRs) activate inflammasomes, which are multiprotein complexes that act as platforms for the activation of proinflammatory caspase-1. Here, PRRs are the cytosolic NLRs. With regard to the innate immune response, cGAS reacts to the presence of mtDNA in the cytosol, initiating the cGAS-STING pathway, which generates a downstream immune response, driving type I IFNs and other proinflammatory cytokines.

In respect to viral infection it was reported that Influenza A, for instance, leads to the dissipation of mitochondrial membrane potential, which ultimately causes mitochondrial fragmentation [23]. Dengue virus (DV) inhibits DRP1 and induces mitochondrial fragmentation, which is necessary for its replication and immune evasion. Thus, targeting mitochondria seems to be an evasion mechanism used by the pathogen in relation to host immunity. It is conceivable that this might lead to mtDNA alterations, then to its release into cytosol or outside the cell, eliciting a counterbalancing inflammatory response by the cell, as described above. The exact mechanism whereby mtDNA can escape into the cytosol remains unknown. At this stage of research, it is difficult to clearly distinguish the effect of the virus’ intracellular targeting of mitochondria from the effect of the release of extracellular mitochondria components. In addition, in respect to the paucity of mitochondria in neutrophils, it seems that mitochondria won’t play a quantitative pivotal role in NETs formation, but could have higher influence with extracellular traps as generated by eosinophils or basophils [2].

### 4.3. RNA and Modified Nucleic Acids

Our understanding of the rules used by innate immune sensors to distinguish ‘self’ from ‘foreign’ remains at an early stage. For RNA, 5′ capping of the terminus is an excellent example, as this feature is present in many viruses, but not in host ribonucleic acid (RNA). As highlighted by Elkon et al. [3], RNA-reactive TLRs can detect both virus and mammalian RNA. TLR7 and TLR8 preferentially bind unmodified uridine (U)–rich single-stranded RNA (ss-RNA) [3]. In addition, epigenetic modifications, such as DNA methylation, were reported as exacerbating chronic airway inflammatory diseases [24]. DNA sequence remains relevant, however, because mammalian DNA sequences enriched for CpG dinucleotides are more potent activators of TLR9 than DNA sequences devoid of CpG dinucleotides [24]. CpG islands, mtDNA, and retroelements are potential sources of immuno-stimulatory DNA in mammals. Double-stranded RNAs were shown to activate nucleic acid-recognizing TLRs, including TLR3; TLRs 7 and 8 are activated by RNAs. It should be noted that NET formation is stimulated by the exposure of neutrophils to certain bacteria, and to immune complexes (ICs) containing RNA, which stimulates the intracellular TLR8, the dominant TLR in neutrophils [3]. Note that viral RNA may be also recognized by MDA5 cellular sensors, which mediate the induction of interferons during viral infection [25].

### 4.4. DNA Re-Entry into Cell

In chronic inflammatory disorders, exacerbated inflammation has been associated with a failure in innate immune tolerance to self-DNA. In physiological conditions, self-DNA released by dying cells is not detected by intracellular DNA sensors. Excessive nucleic acids released by necrosis or NET formation may re-enter the cell. Certain types of nucleic acids can provoke a robust innate immune response, and this recognition is mediated by cytoplasmic receptors such as retinoic acid–inducible gene 1, and by TLRs localized inside endosomes and lysosomes [3,26]. The TLRs recognize a wide array of both endogenous-associated and damage-associated molecular patterns which trigger innate immune responses [27,28]. TLRs can be divided into two categories: those mostly expressed on the cell surface (such as TLR2 and TLR4), and those expressed predominantly in the intracellular compartments, endosomes and lysosomes. TLRs are central to the control of innate immune responses, by their recognition of pathogen-associated molecular patterns (PAMPs) and DAMPs. TLRs are therefore not only critical for host defense against pathogens, but also contribute to the pathogenesis of autoimmunity. These associated proteins may protect the bound nucleic acid from degradation and/or facilitate the associated proteins’ entry into the cell, as is the case for Fc receptor–mediated uptake of antibody-nucleic acid complexes. TLR-mediated pathologic responses to nucleic acids may contribute to other pathologies, such as damage due to liver injury or lung infection, pancreatitis, and graft-versus-host disease. Consequently, re-entry capacity causes nucleic acids to be seen as “ectopic,” foreign invaders, and activates nucleic acid sensors (reviewed in Elkon et al. [3]). Interestingly, Poli et al. [29] discovered that IL-26 binds to genomic DNA, mitochondrial DNA, and NETs, and shuttles them within the cytosol of human myeloid cells.

## 5. NET and Extracellular DNA Release Function: A Double-Edged Sword

As an innate response, NETs formation is on the one hand an efficient strategy for counteracting such intrusive micro-organisms as bacteria and fungi. On the other hand, however, the effects of NETs in high amount on the host can be detrimental, by the fact of the toxicity of its exposed compounds to endothelial cells and to parenchymal tissue [12].

### 5.1. Beneficial Innate Response

The anti-infectious role of NETs, as a new effector mechanism of innate immunity, has been widely documented since their discovery. The unique structure of NETs allows them to capture a wide variety of micro-organisms. Consequently, many examples of NET formation target bacteria appear in the literature, including S. aureus and Mycobacterium tuberculosis. It has also been shown that fungi (*Candida albicans*, *Aspergillus* sp.), Protozoa (*Toxoplasma gondii*) and, more recently, viruses like human immunodeficiency virus (HIV), are sensitive to the antimicrobial action of NETs.

Evidence is accumulating that neutrophils play a role in antiviral immune responses. These virus-induced NETs can both control the virus and damage the host [30].

In vitro experiments have indicated that viruses can directly stimulate neutrophils so that they produce low levels of NETs [30]. For example, neutrophils sense HIV-1 by endosomal PRRs that detect viral nucleic acids, and through mediation of TLR7 and TLR8 subsequently induces NET formation (i.e., influenza virus A). Viruses also induce NET formation indirectly without engaging PRR expression by neutrophils. Thus, NET formation is triggered by the cytokines and chemokines, such as IL-8, contained in the inflammatory milieu created by virus-infected endothelia and epithelia. In addition, type I IFN is abundantly produced during viral infections, and this primes neutrophils to form NETs. Growing evidence suggested that platelets are important for defense against viruses, as platelet are frequently activated during viral infection. For example, ssRNA viruses from the family Picornaviridae family activate platelets through TLR7, reducing viral titers and increasing host survival. Activated platelets aggregate with neutrophils and thereby stimulate NET formation, as reviewed by Schonrich et al. Massive activation of the platelet/neutrophil axis and subsequent NET-based clearance mechanisms may represent a host emergency strategy when facing systemically multiplying viral infections.

Alternatively, there are direct mechanisms by which NETs develop antiviral activity. First of all, the web-like chromatin backbone of NETs can bind to, and immobilize, viral particles due to the attraction between positively charged histones and the negatively charged viral envelope. This mechanically prevents the virus from spreading. Examples include histone H1 binding to noroviruses and H3 and H4 aggregation of seasonal influenza A particles. Second, known antimicrobial molecules against both enveloped and non-enveloped viruses, such as MPO, cathelicidins, and α-defensin, are attached to the chromatin backbone of NETs and can inactivate viral particles.

In addition, NET components indirectly contribute to antiviral immunity by stimulating antiviral effector mechanisms from other immune cells. For instance, HMGB1 proteins anchored in NET fibrin act as DAMPs that trigger the release of pro-inflammatory cytokines and chemokines by other immune cells. Moreover, neutrophil-derived NETs build aggregates that in turn degrade cytokines and chemokines, resulting in self-limitation of the innate immune process. In addition, NETs activate pDCs through TLRs. NETs thus play a key role in antiviral immunity by promoting the release of large amounts of type I IFN. Additionally, NETs can be enriched in oxidized mitochondrial DNA, which efficiently induces a type I IFN response.

### 5.2. Detrimental Innate Response

#### 5.2.1. Sterile Diseases Induced by Unbalanced NET Formation

NETs are not produced exclusively during severe infections. They are also found in non-infectious diseases, in which their presence may be a maladaptive response which leads to tissue injury. NETs and NET by-products have been observed in various non-infectious diseases such as inflammatory diseases, autoimmune diseases and non-autoimmune diseases. These sterile pathologies induced by unbalanced NET formation offer insight into the pathogenesis of COVID-19.

#### 5.2.2. Autoimmune Diseases:

Considerable study has been done on the deleterious role of NETs in the context of autoimmune diseases. The most notable example is systemic lupus erythematosus (SLE), where its deregulation has been repeatedly demonstrated. NETs expose many autoantigens in the extracellular space, in particular double strand DNA or MPO. The immune complexes found in the sera of patients suffering from SLE typically contain nucleic acids associated with various proteins. These include antibodies, the chromatin-associated protein HMGB1, the antimicrobial peptide LL39, ribonucleoproteins, and others [28]. Patients with lupus have increased circulating levels of NETs, and evidence for NET formation can also be found in those patients’ tissue. There is some evidence that patients with SLE have an impaired ability to degrade NET [3]. This protection against DNases leads to a continuous release of IFN, a key cytokine in the pathophysiology of SLE. This cytokine is also responsible for sensitizing PNs to NET formation. The overall result is the establishment of an amplifier loop which may participate in the sustainability of the autoimmune reaction, and in the progression of the disease. NET formation also appears to be dysregulated under diabetic conditions. Hyperglycemic conditions cause oxidative stress and constitutively activate NET formation, which affects the normal immunological balance and also creates microvascular complications [26]. Other autoimmune diseases involving NET formation unbalance are Rheumatoid arthritis (RA), antiphospholipid syndrome, psoriasis, and autoimmune vasculitis rare conditions affecting small blood vessels, particularly those of the lungs, skin and kidneys (Table 1).

#### 5.2.3. Intravascular Diseases:

An increasing number of studies demonstrate the essential role of NETs in thrombotic pathologies, both venous and arterial. The procoagulating effect of NETs is underpinned by their filament structure, which facilitates adhesion and platelet activation. This structure also allows them to capture red blood cells, enabling the formation of the thrombus and the initiation of coagulation. Neutrophils may play a major role in immunothrombosis, which is defined by interactions between innate immunity, inflammation and thrombosis, leading to thrombin generation, especially in microvessels. Fuchs et al. [20] observed that nucleosomes and inflammatory markers are concomitantly elevated in acute thrombotic microangiopathy (TMA). Pertiwi et al. [4] demonstrated that neutrophils and NETs are intricately involved in all types of acute hemorrhagic or thrombotic plaque complications, and that the NETs disperse as the thrombus mass ages. Thus, intravascular NETs may induce platelet trapping and microvascular occlusion leading to multi-organ failure. In the same vein, there is some evidence that patients with vasculitis have an impaired ability to degrade NETs [3], and that cytokines released by neutrophils alter the membrane profile of vascular cells, especially of endothelial cells [5]. Da Cruz et al. [59] revealed that neutrophil elastase (NE)/DNA complexes in NETs play a central role in a mechanism, which results in severe fibrinolytic failure. NE forms a tight complex with DNA that strongly impairs its inhibition by a1-PI [23]. In this way, NE highly degrades plasminogen without generating plasmin, which in turn leads to the production of antifibrinolytic plasminogen fragments. NETs can therefore serve as a platform for NE-mediated activation of intravascular coagulation in vivo [60]. As such, thrombosis and vasculitis appears as one of the physio-pathological hallmarks of NET formation dysregulation (Table 1).

#### 5.2.4. Inflammatory Diseases:

It is likely that the formation of NETs during acute and chronic inflammatory diseases is favored by tissue- and systemic-proinflammatory cytokines. The latter are implicated in multiple-organ failure and increased lethality, for instance in sepsis, acute inflammatory lung diseases, pre-eclampsia, gout, and cystic fibrosis. They are often associated with endothelial damage, tissue ischemia, and NET-induced thrombotic complications. In addition, in humans the presence of NETs has been reported in blood samples from approximately half of patients with severe sepsis. In acute inflammatory lung diseases such as ARDS or post-transfusion acute respiratory syndrome, activated platelets induce the formation of NETs, which accumulate at the alveolar level and participate in epithelial and endothelial lesions (as reviewed by Cheng et al., [12]). In fact, it has been shown that histones are capable of inducing dose-dependent cell apoptosis, and that elastase increases the permeability of the alveolocapillary membrane by alteration of the endothelial cytoskeleton. During post-transfusion acute respiratory syndrome sepsis, circulating markers of NET formation are present. During pre-eclampsia, certain placental factors (i.e., IL-8) are capable of inducing NET formation in vitro. Ex vivo, NETs are abundantly found in the intervening space of the placentas, and may contribute to the hypoxic lesions characterizing this pathology. In gout, it has been shown that sodium urate crystals are capable of inducing the formation of NET, and that these are protected from degradation by the fixation of C-reactive protein (CRP) molecules and of the complement [13]. NETs could therefore persist long enough at the joint to contribute to the establishment of chronic inflammation. In cystic fibrosis, several studies have demonstrated the abundant presence of NETs in the sputum of patients [45]. 

#### 5.2.5. Other Pathologies:

The formation of NETS has also been observed in pathologies such as painful sickle cell crisis, periodontitis, transfusion-related ALI, Felty syndrome, liver diseases (alcohol-associated liver disease and portal hypertension), kidney diseases [21], in severe obesity [57], and in neuropathologies [61] (Table 1).

#### 5.2.6. Conclusion on Sterile Diseases Associated With NETs Non-Balanced Formation and Degradation:

The circulation of detectable amounts of NETs in the serum may impair or overwhelm the NET degradation and clearance machinery. This systemic NET ‘overflow’ has severe harmful effects, both direct and indirect. Firstly, NETs can directly damage the endothelial cells lining the interior face of blood vessels. Secondly, NET overflow drives auto-destructive processes, as NETs components act as neo self-antigens and induce auto-antibodies. In light of this, it may be remarked that a number of molecules identified as important targets in autoimmune diseases (e.g., dsDNA, histones, MPO, vimentin, and enolase) are actually NET constituents. As the result of auto-antibody production, further neutrophil recruitment and the triggering of NET formation, these highly immunogenic NET structures generate a self-perpetuating cycle of autoimmune combat. This is in line with the characteristics of inflammatory lung diseases: exaggerated neutrophil recruitment, activation, and NET formation. The prolonged presence of NETs appears extremely deleterious to host tissue, and can stimulate autoimmune responses due to its high immunogenicity. NETs and NET by-products can shatter self-tolerance in being a source of auto-antigens for auto-antibodies found in autoimmune diseases, and can accelerate the inflammatory processes by releasing a wide range of active molecules, including DAMPs, histones and lytic enzymes such as elastase (as reviewed by Fousert et al. [62]).

## 6. NETs and NETs By-Products: Viral Pathogenesis

The respiratory tract is considered one of the most vulnerable places for microbial invasion of the body. In this context, it is possible that NETs are initially produced in response to pathogens before infection [63]. Caudriller et al. [14] have hypothesized that NET formation occurs in the lungs and is driven by interactions between activated platelets and neutrophils. NETs may produce lung endothelial injury mediated by exposed extracellular histones, neutrophil granular proteins, and by a tangled web of extracellular DNA. This may provide a template for the trapping of platelets and for thrombus formation in the lung microcirculation. NETs induce injury to the lung by a direct toxic effect on endothelial cells; this toxicity may be the result of a high local concentration of histones and granular proteins. The observation of NETs in the lung microvasculature is associated with an increased presence of NET components in the plasma [14].

With regards to viral infection, NETs have been detected in the broncho-alveolar lavage fluid of children with severe respiratory syncytial virus (RSV) infection of the lower respiratory tract. NETs were also detected in dense plugs occluding the small airways in RSV-infected calves. In a mouse model of influenza pneumonia, NET formation was also observed in areas of alveolar-capillary damage in the lung. Hamaguchi et al. [33] have shown that, in response to respiratory infection, neutrophils released NETs abundantly. NET length correlated with six clinical parameters as the explanatory variables: WBC, platelets, lactate, CXC ligand-2, interleukin-8, and procalcitonin.

As mentioned earlier, unbalanced NET formation is associated with pathological conditions, and is directly cytotoxic to epithelial and endothelial cells, as well as to hepatocytes. NET formation represents a plausible link between viruses and systemic autoimmune disease.

It should be noted that transient systemic NET overflow, due to increased NET formation, without noticeable deficiency in DNase-1 activity, can occur during infection with hantaviruses. Neutrophils play an antiviral role during the viral hemorrhagic fevers (VHF) caused by such viruses. In their elegant review, Schonrich et al. [30] describe how these zoonotic pathogens belong to the Bunyaviridae family, and infect humans after transmission via the inhalation of aerosolized urine, saliva, and feces from chronically-infected rodents, who are their natural hosts. In humans, these pathogens can induce severe pulmonary and renal dysfunction, as well as intravascular coagulation and hemorrhagic shock. High levels of circulating NETs, and accordingly increased amounts of cirDNA and histones, are detected in hantavirus-infected patients. Thrombin generation, intravascular coagulation and increased endothelial barrier permeability feature among other hantavirus infection effects. This is in line with the potential detrimental effects of excess NET formation. Finally, NETs can induce auto-antibodies which may contribute to the systemic pathology of hantavirus-associated disease. Neutrophils can detect HIV-1 via interaction with TLRs, especially TLR7 and TLR8. These TLRs recognize viral nucleic acids and induce the generation of reactive oxygen species by MPO-derived oxidants that trigger NET formation and elimination of HIV-1. This response may, however, be counteracted by HIV-infected dendritic cells which release IL-10, an anti-inflammatory cytokine; that in turn inhibits NET formation. Notably, it was also demonstrated that viral infections with rotavirus RV and RSV alter DNA methylation and histone modifications in the airway epithelium. This may in turn alter inflammatory responses, exacerbating chronic airway inflammatory diseases [24]. Furthermore, NETs and neutrophils are involved in the pathologies of chikungunya virus (CHIKV), simian immunodeficiency virus (SIV), influenza, parvovirus, rhinovirus, and influenza-associated pneumonia.

Elastase-mediated activation of SARS-CoV2 was originally reported by Taguchi and co-workers [64], and the potentially significant implications of elastase for viral pathogenesis has been proposed (6,7). NE has been found to be responsible, at least partially, for an increase of the severity of SARS-CoV2-mediated infection, by means of multiple mechanisms (6,7): (i), like other proteases (trypsin, TMPRSS2, or plasmin), NE enables S protein cleavage and SARS-CoV2 entry into cells directly from the cell surface [61]; (ii), NE is a known activator of epithelial sodium channel (ENaC) subunits; this results in Na+ hyperabsorption, reduced airway surface liquid height, and dehydrated mucus, culminating in inefficient mucociliary clearance (such as found in cystic fibrosis); (iii), NE is also known as one of the most destructive enzymes in the body [65]. An overwhelming release of enzymatically active elastase, together with simultaneously produced ROS, can cause local tissue injury.

Lastly, a small but growing number of cases of "multisystem inflammatory" disease in children that required intensive care have been reported. Symptoms include fever, severe abdominal pain and skin rashes, along with markers of severe inflammation in the blood, including some instances of heart inflammation. The clinical conditions are common with those of Kawasaki disease, and we inferred a link with COVID-19 from the fact that a significant fraction of these children were tested positive for COVID-19 [66]. Yoshida et al. [67] recently reported that spontaneous NET formation was enhanced in the neutrophils of patients with acute Kawasaki disease. This association supports our hypothesis that NETs and NET by-products play a key role in COVID-2 pathogenesis [68].

## 7. Strategies in Targeting NET Formation and NETs By-Products

A disproportionate virus-induced NET release can contribute to physiological damage, locally as well as systemically. It is therefore important to explore the mechanisms that control NET formation in the context of viral infection. Here, we first focus on two strategies targeting [1] the physical effect of NETs by DNase1; and [2], the proteolytic activity of neutrophil elastase.

### 7.1. DNase1

Naturally-occurring DNase-1 digests extracellular chromatin and NETs [14,28,69,70]. Low level bioactivity of endogenous DNase-1 may lead to a dysregulation of NETs, thus causing autoimmune diseases and other inflammatory disorders. DNase-1 is the only NET-targeting molecule already in use in clinical practice, being used to treat both cystic fibrosis (in order to improve lung function and reduce the occurrence of infectious exacerbations) and virus-associated bronchiolitis [20,21]. However, the fact that DNase-1 dismantles the NET structure without degrading the whole protein components of NETs, indicates that it would be less effective in abrogating a NET-triggered inflammatory response. Such a response can, however, be targeted by the use of histone-blocking antibodies [9]. There are ongoing clinical trials evaluating DNase1 as a COVID-19 treatment [71,72]. One possible drawback in using DNase1 could be the subsequent release of granule proteases, such as NE, potentially causing cytotoxicity. Consequently, vascular examination would appear to be necessary in cases of acute exacerbation of NET formation.

### 7.2. Neutrophil Elastase

The multiple mechanisms of NET-associated elastase have various effects as we recently reported [73]: they directly affect SARS-CoV2 entry to cells, and indirectly cause thrombosis, coagulopathy, hypertension, and endothelial and epithelial tissue injury [73]. In addition, the clinical outcome of patients with COVID-19 may be improved by preventing (or at least reducing) the entry of SARS-CoV2 into respiratory cells, and reducing fibrinolysis or hypertension by NE. Note that the use of Azithromycin in combination with hydroxychloroquine (HCQ) to treat COVID-19 showed a significant decrease in NE. Consequently, in light of the COVID-19 physiopathology. The inhibition of this serine protease would appear to be a good therapeutic option. Taken together, this strongly suggests the promise of treatments targeting elastase, such as the use of the small molecule inhibitors—Sivelestat [74], Alvelestat or Bay-8550 [75].

### 7.3. Other Strategies

As regards neutrophil/platelet interactions, it should be noted that aspirin treatment decreases NET formation in the lung microcirculation and plasma, and also decreases the deposition of platelets with neutrophils on the lungs’ vascular walls [9]. Very different structural classes of molecules can inhibit the potent neutrophil stimulus for the release of NETs by platelet activation of endosomal TLRs 10. Such approaches include anti-CLEC [28], and especially a bispecific anti-CLEC5A/TLR2 monoclonal antibody [70]. Hydroxychloroquine (HCQ), a widely-used anti-malarial and anti-inflammatory drug, shows TLR-pathway blockage capacity [76]. The use of biologics to block cytokines is now widespread, as in the use of newer, small molecule drugs such as ‘Jakinibs’ [77], or anti-interleukin 6 (IL-6) or anti-interleukin 1 (IL-1) therapies to block neutrophil function [78]. Given the importance of the mechanism of self-DNA re-entry allowed by IL-26, the possibility of targeting this other cytokine must be considered [29]. The antidiabetic drug Metformin directly binds the alarmin HMGB1, resulting in higher NETs clearance and leading to inhibition of NETs’ pro-inflammatory activity [55,68,79]. Glycocorticoides such as dexamethasone is a class of drugs with anti-NET formation activity [80]. Lastly, the identification of endogenous NET inhibitory peptides suggests their potential as therapeutic agents [81].

## 8. Viral-Induced ARDS/Cytokine Storm

In this section, we focus on the link between the cytokine storm and ARDS, as they combine to cause severe illness in the case of COVID.

It is well known that most pro-inflammatory cytokines are released from macrophages. Severe acute infections are usually associated with the activation of macrophages by enveloped viruses such as the DV, H5N1 viruses, and Ebola viruses [66]. As indicated above, the activation of neutrophils leads to the formation of NETs, which may further aggravate virus-induced inflammatory reactions. It is well established that acute viral infections frequently cause thrombocytopenia, and that platelet-leukocyte interactions not only regulate inflammatory reactions but also contribute to the pathogenesis of vascular injury, thrombosis, and autoimmune reaction [76].

Whilst the host immune system is beneficial in promoting the clearance of many pathogens, it can also have adverse effects in certain contexts [20]. One of the most cited examples is the high susceptibility to bacterial infection after acute viral infections, for instance with the influenza virus. This may be due to the association of NET formation with lung injury, and the central role of inflammasome activation in viral pathology.

Invading viruses mediate the activation of platelets, resulting in the enhancement of NET formation and pro-inflammatory cytokines release; it also induces inflammasome activation via membrane receptors, including CLECs. Sung et al. [70] recently showed that the blockade of CLEC5A/TLR2 attenuates NET formation and inflammasome activation, and is thus beneficial to the host during acute viral infection. CLEC5A/TLR2 is therefore critical in microbe-induced NET formation and pro-inflammatory cytokine production. In particular, these effects are induced by DV and H5N1 influenza virus, by the release of extracellular vesicles; these vesicles can be regarded as novel DAMPs, which activate innate immunity via CLEC5A and TLR2.

Respiratory failure from ARDS is the leading cause of COVID-19 associated mortality. Accumulating evidence suggests that a subgroup of patients with severe COVID-19 might suffer from cytokine storm syndrome. The phenomenon known as a “cytokine storm” corresponds to a massive over-production of cytokines by the host immune system. In a recent retrospective multi-center study of 150 confirmed COVID-19 cases in Wuhan, China, elevated ferritin and IL-6 were found as predictors of lethality, suggesting that mortality might be due to virally-driven hyperinflammation. The cytokine storm was formerly observed and investigated during the global outburst of SARS-CoV infection in 2003. That virus results in a severe inflammatory reaction, and increases systemic vascular permeability, which occurs within two to five days following viral exposure.

Almost 200,000 persons in the US develop ALI every year from a variety of causes, including sepsis, bacterial pneumonia, aspiration of gastric contents, and epidemic viruses such as H1N1 and SARS [14]. NETs have been found in infection-related ALI models of influenza virus, bacteria or bacterial component LPS, and fungi. Since the 1980s, substantial inflammation in the alveolar and plasma compartments has been a recognized as characteristic of patients with ARDS. High concentrations of inflammatory cytokines (i.e., the result of a cytokine storm) have been associated with both injurious ventilator strategies and poorer outcomes. During a cytokine storm, an excessive immune response ravages healthy lung tissue, leading to acute respiratory distress and multi-organ failure. Beyond inflammation, mechanisms established as major contributors to ARDS pathogenesis include endothelial activation, respiratory epithelial dysfunction, and surfactant depletion.

It has been discovered that the activation of endosomal TLRs (TLR3, TLR7, TLR8, TLR9) and cytosolic sensors by viral nucleic acids induces the production of interferons and proinflammatory cytokines. These intracellular nucleic acid receptors/sensors have been defined as “protective host factors,” being critical for host defense against viral infections. However, the identity of “pathogenic host factors,” and their specific contribution to virus-induced severe inflammatory reactions and lethality, remains unclear. Moreover, the origins of distinct clinical symptoms as provoked by different viruses have yet to be fully elucidated.

Indeed, the most common critical complications during exacerbation of COVID-19 are ARDS, sepsis, acute cardiac injury, heart failure, systemic vascular permeability, disseminated intravascular coagulation, and venous thromboembolism. NET formation dysregulation observed in severe cases tends to be characterized by lower lymphocytes counts, higher leukocytes counts, and a higher neutrophil-lymphocyte-ratio [82], as well as lower percentages of monocytes, eosinophils, and basophils. Biologics such as high levels of IFN, lactate deshydrogenase, CRP, and fibrinogen are reported in a minority of cases.

## 9. Proposed Mechanisms for the Role of NETs Formation in COVID-19

The early onset of inflammatory reactions with elevated local or systemic vascular permeability, are the common features of SARS-CoV2 and other acute viral infections prior to the adaptive immune system being fully activated. Severe COVID-19 occurs in the second week after the debut of symptoms, in contrast to other virus infection, such as flu. These observations suggest that innate immune responses contribute significantly to the pathogenesis of acute viral infections [83]. Considering the rapid evolution of the symptoms and the lethality of this disease, we hypothesize that COVID-19 progresses under an uncontrolled amplifier loop leading to hyperinflammation. Refractory patients generally present cytokine storm syndromes sharing the pathology of an overly active immune response, frequently leading to fatal multi-organ dysfunction syndrome, including ARDS.

We were among the very first to draw a possible link between COVID-19 and NETopathies by reporting examples of the correlations of COVID-19 symptoms with those consecutive to uncontrolled NET formation causing various sterile or infectious diseases [68,84,85]. Here, we describe all the common general clinical conditions, and numerous pathological and biological features with NET deleterious effects (Table 2). We postulate that COVID-19 induces a disproportionate virus-induced NET release, which plays a key role in COVID-19 pathogenesis.

Viruses and notably human respiratory RNA viruses, are known for their extraordinary capacity to evade immune control mechanisms [83]. We postulate that viral mechanisms target NET formation by impairing the clearance of NET and extracellular DNA [86]. This would lead to harmful positive amplification of virally-driven hyperinflammation. This finding suggests a significant new direction for the development of treatments for this acute viral infection. Investigation of NET formation and the pathogenesis of extracellular DNA is all recent, with the result that research and development on treatments that would inhibit the amplifier loop induced by unbalanced NET formation also remains in its infancy. While neutrophils are the principal starting point for DNA release, targeting NETs rather than neutrophils themselves may in practice be a preferable strategy.

Two main phases might be considered in the progression of COVID-19: at the appearance of the first symptoms, and again at the start of the cytokine storm featuring respiratory failure. While antivirals can be administered throughout the course of the illness, the use of immunomodulators appears less beneficial and even counterproductive, at the point where the immune response is exacerbated, possibly due to NET formation’ “double-edged sword” effect. COVID-19 mild disease should feature an early onset of inflammatory reactions with elevated local or systemic vascular permeability, before the adaptive immune system is fully activated, highlighting the significant contribution of the innate immune response. This is the case for the majority of patients infected with COVID-19, who experience only mild or no symptoms in the course of the disease. In this light, the COVID-19 evolution may not be unique. Nonetheless, it is obviously a complex disease, since a minority of infected patients suffer severe or lethal conditions, and since the apparent SARS-CoV2 genomic stability is observed up to date.

Our belief is that the cytokine storm, and the downstream local and systemic cytotoxicity to endothelial and epithelial walls, result from the impairment of NET and cirDNA. We suggest that SARS-CoV2 induces a disproportionate virus-induced NET release, and that this plays a key role in the COVID-19 pathogenesis. Furthermore, it appears to us likely that these patients possess pathogenic host factors which permit SARS-CoV2 to evade an innate immune response, which may in turn incur chronic NET auto-stimulation, with an impact similar to that of an autoimmune disease [60].

We hypothesize that this adverse, self-inflicted pathological effect, could almost certainly due to host factors in critically ill COVID-19 patients [87]. Those with the greatest risk of developing a serious form of COVID-19 are older adults or people with existing chronic medical conditions [1,88,89] (Table 1). In a recent report considering elderly residents with COVID-19 in a long-term care facility [31], the most prevalent chronic underlying health conditions present were hypertension, diabetes mellitus, cardiac, renal and pulmonary diseases, or obesity (Table 1). These conditions correlate with the pathologies resulting from NET formation dysregulation, as described above. Given that context, it is obviously necessary to investigate the influence of comorbidities, individual immune response, previous or co-infections, previous diseases, ethnicity, and genetic or epigenetic alterations on COVID pathogenesis and NETopathies [87].

Recent findings have revealed that some ethnicities are more likely to develop serious COVID-19, in particular with respect to thrombotic risk [87]. For instance, Chinese patients experienced a three to four fold lower incidence of venous thromboembolism than Caucasians [90]. In addition to the unexplained differences in coagulopathy, this indicates possible racial susceptibility to COVID-19. In view of the variations in host immunological and inflammatory responses, individual predispositions of various natures characterize COVID-19 physiopathology. We recently described possible COVID-19 host/genetic factors [87]. As we postulate that the exacerbation of NET formation plays a key role in COVID-19 pathogenesis, we also described a number of genetic factors affecting NET formation upon stimulation [87]. These may contribute to the as yet unexplained differences which are beginning to be identified concerning ethnic susceptibility to COVID-19 mortality. As advances continue to be made in the understanding of COVID-19 pathogenesis, deciphering genetic predispositions might in the future lead to the development of precision medicine, should the pandemic remain a serious public health issue.

The high incidence of thromboembolic events from autopsy findings suggests an important role of COVID-19–induced coagulopathy [91,92,93,94]. In addition to the main strategies such as targeting SARS-CoV-2 attachment and entry to human and viral replication or systemic immune responses, targeting hyper-inflammation with steroids, such as dexamethasone and non-steroidal anti-inflammatory drugs (NSAIDs) have been attempted in a number of clinical trials [95]. Among NSAIDs, the anti-platelet drug, aspirin is significantly associated with reduced risk of developing COVID-19 certainly due to anti-inflammatory and anti-coagulant effects [95]. Thus, we speculate that aspirin may reduce NETs induced immuno-thrombotic events, and that observation of NETs formation in the course of COVID-19 clinical trials involving aspirin administration that are ongoing, would be of interest.

A small fraction of COVID-19 patients experienced after recovery and viremia absence still some mild after side effect such as pain, fatigue or slight neuropathies. One could put into consideration such sequelae from the disease as a challenging medical issue. As NETopathies appears involved in COVID-19, this highlight the need of studying unbalanced NETs formation or of evaluating treatment by NETs inhibitors following survivors’ recovery. Of the various pathological sequelae caused by [NETs and NETs by-products] following an adaptive immune response in patients with prior severe illness, we have highlighted here those caused by NE, believing them to be the most deleterious/significant [73]. We postulate that sequelae are mostly due to systemic coagulopathies and vascular walls deterioration, resulting, in particular, from exacerbated NET formation and NE released from NET disintegration, respectively [87].

The correlation of NET formation in COVID-19 with clinical and pathological parameters describing the severity of disease has yet to be analyzed in larger cohorts. Moreover, therapeutic strategies aiming at down-regulating NET formation or increasing NET disintegration will be tested in clinical trials. Treatments currently in use or in the final stages of clinical development include: Remdesivir (GS-5734), a Lopinavir/Ritonavir association (with or without Interferon 1ß), HCQ, anti-IL-6, Jakinibs or intravenous immunoglobulin are evaluated to treat COVID-19 [77]. So far, these treatments have shown no major effects in clinical trials (Table 3). Considering the COVID-19 pandemic and its severe impact on public health, the clinical imperative now should be to implement monotherapy or combination therapy alongside other options. We firmly believe that clinical research into compounds showing an effect on NET formation deserves to be assessed. We propose that, in the short term, DNase-1 treatment should be evaluated in clinical trials, with or without associated drugs. Anti-elastase drugs such as Sivelestat or anti-diabetic drugs such as Metformin (Table 3) could also be assessed as part of a fast-track drug repurposing strategy for treating COVID-19 [96]. In the longer term, we also propose a significant increase in research on the development of TLR and CLEC inhibitors, NET-inhibitory peptides [93], and on anti-IL-26 therapies.

## 10. Conclusions

This review points out the key role of NETs formation in COVID-19 pathophysiology highlighting associated risk factors and new potential therapies. Several clinical studies are ongoing to corroborate this notion with more experimental data. A better understanding of extracellular traps in COVID-19 pathogenesis would not only provide therapeutic options to reduce SARS-Cov2 viral replication as well as to improve patient survival, but also potentially turn down sequelae outcome.

## Figures and Tables

**Table 1:** Legend: comparison of neutrophil extracellular trap (NET) dysregulation associated diseases and coronavirus disease-2019 (COVID-19) comorbidities/host risk factors.

Diseases	NET Dysregulation Associated Diseases	COVID-19 Comorbidities/Host Risk Factors
**Non-autoimmune**		Cerebrovascular disease * (McMichael et al. [31])
	**Acute lung injury (Grommes et al. [32])**	Pulmonary diseases * (McMichael et al. [31])
	**SIRS (Hamaguchi et al. [33])**	
	**Sepsis (Chen L et al. [34])**	Sepsis (Hirose et al. [9])
	**Thrombosis (Frangou et al. [35])**	
	**Kidney diseases (Nakazawa et al. [21])**	Kidney diseases * (McMichael et al. [31])
	**Obesity (D’abondanza et al. [36])**	Obesity * (McMichael et al. [31])
	**Vasculitis (Kessenbrock et al. [37])**	
	**Sickle cell disease (Schimmel et al. [38])**	Sickle cell disease (Hussain et al. [39])
	**heart failure (Bonaventura et al. [40])**	Cardiovascular disease * (McMichael et al. [31])
	Atherosclerosis (Doering et al. [41])	
	**hypertension (Vijay et al. [42])**	Hypertension * (McMichael et al. [31])
	**Disseminated intravascular coagulation (Tang et al. [43])**	Disseminated intravascular coagulation (Stiel et al. [5])
	**Chronic inflammation disease (Tan et al. [24])**	Chronic inflammation disease (Yang et al. [44])
	Cystic fibrosis (Manzenreiter et al. [45])	
	TRALI (Caudriller et al. [14])	
	**Periodontis (Lee and Lee, [46])**	
	Pre-eclampsia (Moodley et al. [47])	
	***Neuropathy* (Takeuchi et al. [48])**	
	***Gouty arthritis* (Chatfield et al. [49])**	
	Felty syndrome (Dwivedi et al. [50])	
	*Inflammatory bowel disease* (Agelidou et al. [51])	Inflammatory bowel disease (Danese et al. [52])
	Liver disease (Hilscher et al. [53])	
**Autoimmune**	Anti-phospholipid syndrome (Lee et al. [54])	
	Type 1 Diabetes (Menegonza et al. [55])	Type 1 Diabetes (Yang et al. [44])
	
	Psoriasis (Lee et al. [54])	
	**Rheumatoid arthritis (Rohrbach et al. [56])**	*Rheumatoid arthritis* (Favalli et al. [57])
	Lupus (SLE) (Lamphier et al. [28]; Boelz et al. [58])	

* Reported as independent factors as previously reported [31]; reported comorbidity trends in line with NETs dysregulation (bold); comorbidity observed as suggested in line with NETs dysregulation (italic); data updated as for 22 April 2020.

**Table 2:** Common pathological conditions or biological features shared by NETosis/extracellular DNA dysregulation and COVID-19 in severe cases.

Overall	Pathologies	Vascular and Coagulation Consequences	Biological Features
Complex disease	Respiratory failure	Disseminated intravascular coagulation	High level Neutrophils
Inflammatory disease	ARDS	Endothelium damage	High level Interferon
Multi-organ damage	Heart failure	Systemic vascular permeability	High level C reactive protein
	Acute cardiac injury	Prothrombotic	High level Lactate deshydrogenases
	Vasculitis	Abnormality of coagulation function	High level proinflammatory cytokines
	Type 1 Diabetes sensitization		Elevated presence of fibrinogen
	Kidney diseases		High level antiphospholipid antibodies
	Inflammatory bowel disease		
	Chronic inflammation disease		
	Sepsis		
	Rheumatoid arthritis		
	Neuropathy		
	Gouty arthritis sensitization		

**Table 3:** Comparison of COVID-19 studied treatments and proposed NETopathies treatments.

	Main Drugs/Treatments	Biological Targets	Mode of Action
**COVID-19**	Convalescent plasma	virus	virus neutralization
	Monoclonal antibody	virus proteins	virus neutralization
	anti-IL6	IL-6	immuno-modulation
	IFNs	Immunological cells	antiviral proteins synthesis and immunological cell activation
	Jakinib	Janus kinase	immuno-modulation
	Remdesivir	RNA polymerase	inhibition of viral replication
	Ribavirin	RNA polymerase	inhibition of viral replication
	Sofosbuvir	RNA polymerase	inhibition of viral replication
	Lopinavir/Ritonavir	3-chymotrypsine like protease activity	inhibition of viral replication
	Arbidol	virus/cell binding complex	virus penetration into cells
	Camostat mesylate	S protein	virus penetration into cells
	Hydroxychloroquine	ACE2 glycosylation and phagolysosomes	cell penetration and intracellular virus uncoating
	Dexamethasone	Glucocorticoid receptor	anti-inflammatory
**COVID-19 NETopathies**	DNase1	DNA	DNA degradation
	Silvelestat	Neutrophil elastase	antiprotease
	Monoclonal antibodies	histones	histone blocking
	Monoclonal antibodies	CLEC	inhibition of platelet activation
	Monoclonal antibodies	TLR	inhibition of platelet activation
	Monoclonal antibodies	IL-6	inhibition of neutrophil function
	Monoclonal antibodies	IL-1	inhibition of neutrophil function
	Monoclonal antibodies	IL-26	inhibition of DNA self-entry
	Anti-coagulant	Platelet/Neutrophil/NET complex	Inhibition of platelet activation
	Hydroxychloroquine	NET	inhibition of NET stimulation
	Metformin	Alarmin HMGB1	NET clearance
	Colchicin	Neutrophil	neutrophil recruitement

## Abbreviations

COVID-19	Coronavirus 2019 disease
NETs	neutrophil extracellular traps
SARS CoV2	severe acute respiratory syndrome-coronavirus 2
TLR	toll-like receptors
CLEC	C-type lectin like receptors
CDC	Center for Disease Control and Prevention
WHO	World Health Organization
ARDS	acute respiratory distress syndrome
ALI	acute lung injury
PAF	platelet activating factor
IL-8 or CXCL8	interleukin-8
LPS	lipopolysaccharide
PKC	protein kinase C
PMA	phorbol myristate acetate
PAD4	peptidyl arginine deiminase 4
MPO	myeloperoxidase
NE	neutrophil elastase
ETs	extracellular traps
TNF α	tumor necrosis factor α
Cit-H3	citrullinated histone H3
SIRS	systemic inflammatory response syndrome
WBC	white blood cells
cirDNA	circulating DNA
HMGB1	high mobility group box-1
ROS	reactive oxygen species
GM-CSF	colony-stimulating factor
DAMPs	damage-associated molecular patterns
PRR	pattern recognition receptors
STING	stimulator of interferon genes
pDCs	plasmacytoid dendritic cells
ORF-9b	open reading frame 9b
DRP1	dynamin-related protein 1
iIFN	inducible interferon
NLRP3	NOD-, LRR- and pyrin domain-containing protein 3)
NLRs	(NOD)-like receptors
NOD	nucleotide-binding oligomerization domain
DV	dengue virus
RNA	ribonucleic acid
ss-RNA	single-stranded RNA
ICs	immune complexes
PAMPs	pathogen-associated molecular patterns
HIV	human immunodeficiency virus
SLE	systemic lupus erythematosus
RA	rheumatoid arthritis
TMA	thrombotic microangiopathy
CRP	C-reactive protein
RSV	respiratory syncytial virus
VHF	viral hemorrhagic fevers
CHIKV	chikungunya virus
SIV	simian immunodeficiency virus
ENaC	epithelial sodium channel
HCQ	Hydroxychloroquine
IL-6	interleukin 6
